# Mitochondria-Derived Vesicles, Sterile Inflammation, and Pyroptosis in Liver Cancer: Partners in Crime or Innocent Bystanders?

**DOI:** 10.3390/ijms25094783

**Published:** 2024-04-27

**Authors:** Flora Guerra, Francesca Romana Ponziani, Ferdinando Cardone, Cecilia Bucci, Emanuele Marzetti, Anna Picca

**Affiliations:** 1 Department of Biological and Environmental Sciences and Technologies, Università del Salento, Via Provinciale Lecce–Moteroni 165, 73100 Lecce, Italy; flora.guerra@unisalento.it; 2 Fondazione Policlinico Universitario “Agostino Gemelli” IRCCS, L.go A. Gemelli 8, 00168 Rome, Italy; francescaromana.ponziani@policlinicogemelli.it (F.R.P.); luca.cardone27@gmail.com (F.C.); emanuele.marzetti@policlinicogemelli.it (E.M.); 3 Department of Experimental Medicine, Università del Salento, Via Provinciale Lecce–Moteroni 165, 73100 Lecce, Italy; cecilia.bucci@unisalento.it; 4 Department of Geriatrics, Orthopedics and Rheumatology, Università Cattolica del Sacro Cuore, L.go F. Vito 1, 00618 Rome, Italy; 5 Department of Medicine and Surgery, LUM University, SS100 km 18, 70010 Casamassima, Italy

**Keywords:** DAMPs, extracellular vesicles, gasdermin, hepatocellular carcinoma, immunotherapy, immune checkpoints, interleukin, miRNAs, mtDNA, mitochondrial-derived vesicles

## Abstract

Alterations in cellular signaling, chronic inflammation, and tissue remodeling contribute to hepatocellular carcinoma (HCC) development. The release of damage-associated molecular patterns (DAMPs) upon tissue injury and the ensuing sterile inflammation have also been attributed a role in HCC pathogenesis. Cargoes of extracellular vesicles (EVs) and/or EVs themselves have been listed among circulating DAMPs but only partially investigated in HCC. Mitochondria-derived vesicles (MDVs), a subpopulation of EVs, are another missing link in the comprehension of the molecular mechanisms underlying the onset and progression of HCC biology. EVs have been involved in HCC growth, dissemination, angiogenesis, and immunosurveillance escape. The contribution of MDVs to these processes is presently unclear. Pyroptosis triggers systemic inflammation through caspase-dependent apoptotic cell death and is implicated in tumor immunity. The analysis of this process, together with MDV characterization, may help capture the relationship among HCC development, mitochondrial quality control, and inflammation. The combination of immune checkpoint inhibitors (i.e., atezolizumab and bevacizumab) has been approved as a synergistic first-line systemic treatment for unresectable or advanced HCC. The lack of biomarkers that may allow prediction of treatment response and, therefore, patient selection, is a major unmet need. Herein, we overview the molecular mechanisms linking mitochondrial dysfunction, inflammation, and pyroptosis, and discuss how immunotherapy targets, at least partly, these routes.

## 1. Introduction

Hepatocellular carcinoma (HCC) is the fourth most frequent cause of cancer-related death globally and the sixth most common cancer. Among the etiopathogenic mechanisms of HCC, both viral and non-viral causes have been identified [1]. Regardless of the nature of the insult, altered cellular signaling, chronic inflammation, and tissue remodeling are pivotal contributors to HCC [2]. The two main pro-tumorigenic mechanisms through which immune cells promote HCC include the secretion of cytokines and growth factors that either favor proliferation or block apoptosis of cancer cells, as well as, paradoxically, blunt anti-tumor functions of lymphocytes [3]. The nuclear factor κB (NF-κB) and Janus kinase (JAK) signal transducer of activation (STAT) pathways have been well characterized and indicated as key inflammatory routes involved in the promotion of HCC [4].

Aside from canonical routes of systemic inflammation, chronic sterile inflammatory pathways arising from the bulk release of molecules upon tissue injury, collectively indicated as damage-associated molecular patterns (DAMPs), have also been attributed a role in HCC [5]. Cell-free circulating DAMPs have been widely described in HCC; however, cargoes of extracellular vesicles (EVs) and/or EVs themselves can exert similar functions [5] but have been only partially investigated in HCC. For instance, circulating mitochondrial DNA (mtDNA), which is a DAMP itself, can be released within EVs, referred to as mitochondria-derived vesicles (MDVs), and trigger sterile inflammation [5,6,7]. Nonetheless, very little is known about the role of EVs and, in particular, of MDVs in the pathogenesis and progression of HCC. During their generation, EVs can encapsulate a vast repertoire of molecules (e.g., mRNAs, proteins, miRNAs, long non-coding RNAs (lncRNAs), metabolites, lipids, DNA fragments). As such, EVs may represent Pandora boxes holding the advantage of capturing the dynamics of live processes that could be exploited to shed light onto relevant molecular pathways via deep characterization of shuttled molecules. EVs are secreted as a heterogeneous population and by several liver cells, including hepatocytes, stellate cells, and immune cells, to mediate physiological communication and ensure organ homeostasis [8]. Previous studies have described the involvement of EVs in HCC growth, metastasis, angiogenesis, and immunosurveillance escape [9]. However, a current challenge in HCC biology is the comprehension of the molecular mechanisms regulating tumor onset and progression and the identification of biomarkers for early disease diagnosis and progression and of druggable pathways.

Pyroptosis, the caspase-dependent trigger of systemic inflammation via apoptotic cell death, emerged as a relevant player in tumor immunity that may help capture additional details on the molecular pathways sustaining HCC development with a potential predicting power of the prognosis of HCC [10].

Besides traditional clinical management of HCC, the combination of atezolizumab and bevacizumab (atezo-bev) targeting, respectively, the immune checkpoint programmed death-ligand 1 (PDL1) and the angiogenic vascular endothelial growth factor (VEGF) has recently been approved as a first-line systemic treatment for unresectable or advanced HCC [11]. Although, the synergistic anti-cancer effect conveyed by this therapeutic strategy has been successful in some instances, the lack of predictive biomarkers that may allow identification of responders and non-responders is an unmet need [12].

Herein, we overview the molecular mechanisms linking mitochondrial dysfunction, pyroptosis, and inflammation and discuss how immunotherapy targets, at least partly, these routes. A brief discussion of the unmet need in HCC treatment and the molecular pathways that may be exploited as new targets for treatment is also provided.

## 2. Molecular Mechanisms Linking Mitochondrial Dysfunction and Inflammation

Cell death is the result of two distinct processes: (1) apoptosis, also known as programmed cell death; and (2) necrosis, uncontrolled cell death. Alternative forms of programmed cell death exist and, among them, pyroptosis was first identified in macrophages infected by Salmonella or Shigella and not observed in caspase-1 deficient cells. Pyroptosis is proinflammatory, although, like apoptosis, it follows a programmed series of caspase-dependent events [13].

### 2.1. Mechanisms of Cell Death: Apoptosis Versus Necrosis

**Figure 1:**
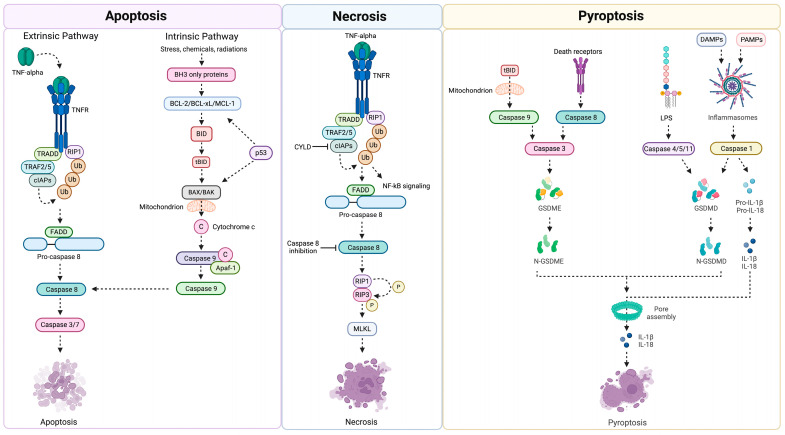
Schematic representation of the main pathways triggering apoptosis, necrosis, and pyroptosis. Abbreviations: Apaf-1, apoptotic protease activating factor 1; BAK, Bcl-2 homologous antagonist/killer; BAX, Bcl-2-associated X-protein; BH3, Bcl-2 homology-3 domain; BID, BH3 interacting-domain death agonist; cIAPs, cellular inhibitor of apoptosis proteins; CYLD, cylindromatosis; DAMPs, damage-associated molecular patterns; FADD, FAS-associated death domain; GSDM, gasdermin; IL, interleukin; LPS, lipopolysaccharide; MLKL, mixed lineage kinase domain-like protein; NF-kB, nuclear factor-kappa B; PAMPs, pathogen-associated molecular pattern molecules; RIP1, receptor interacting protein kinase 1; tBID, truncated BID; TNF, tumor necrosis factor; TNFR, tumor necrosis factor receptor; TRADD, TNFR1-associated death domain protein; TRAF, TNFR associated factor; Ub, ubiquitin. Created with BioRender.com (accessed on 24 April 2024).

Apoptosis is a noninflammatory form of programmed cell death that is executed via intrinsic and extrinsic pathways characterized by the activation of different apoptotic classes of caspases [14]. Mitochondrial damage is the main factor of the intrinsic pathway activation with release of cytochrome *C* into the cytoplasm, and formation of a complex (apoptosome) with the apoptotic protease activating factor-1 (Apaf-1) and the precursor of caspase-9 [15]. The apoptosome triggers the activation of caspase-9 with subsequent engagement of caspase-3/7 leading to cell death by cleavage of different cellular substrates (Figure 1).

The binding of death receptors with specific signals at the cell surface, such as tumor necrosis factor-α (TNF-α), activates the extrinsic apoptotic pathway. This occurs via death receptor oligomerization and recruitment of caspase-8, which directly cleaves the apoptotic effector pro-caspase-3 [16,17]. Caspase-8 also cleaves BID, a member of the BCL-2 (B-cell lymphoma 2) family, to produce a truncated fragment (tBID). The latter, after migration to the mitochondrion to form BAX/BAK pore on its surface, causes the release of cytochrome *C* and subsequent activation of apoptosis through the intrinsic pathway. Hence, caspase-3 activation occurs at the end of both the intrinsic and extrinsic pathway and is a key step in the execution of apoptosis [18,19]. Biochemical and morphological changes of apoptosis, such as membrane blebbing, chromatin condensation, DNA cleavage, and exposure of phosphatidylserine on the extracellular side of the plasma membrane are consequences of the activation of caspase-3 [20] (Figure 1).

Unlike apoptosis, necrosis is an uncontrolled form of cell death that is induced by external injury, such as hypoxia or inflammation, and is characterized by organelle and cell membrane destruction, often accompanied by an inflammatory reaction [21,22]. Upregulation of various proinflammatory proteins and compounds is involved in this process. In particular, the rupture of the cell membrane and consequent spillage of cell content into surrounding areas result in an inflammatory cascade and tissue damage consequent to upregulation of nuclear factor-κB (NF-κB). Another significant difference with apoptosis is that necrosis is an energy-independent process, during which the cell is unable to function as the consequence of severe damage by a sudden shock (e.g., radiation, heat, chemicals, hypoxia). Thus, the cell undergoes a process known as oncosis characterized by swelling. Several studies have shown that gene regulation is also involved during necrosis. A study in *Caenorhabditis elegans* showed that necrosis is mediated by genes encoding plasma membrane and Ca^2+^ channels of the endoplasmic reticulum [23]. Moreover, cyclophilin D, which resides within the mitochondrial matrix [24,25,26], regulates mitochondria-guided necrosis. The mitochondrial permeability transition pore (MPTP), composed of the voltage-dependent anion channel, adenine nuclear translocator, cyclophilin D, and other molecules, plays an important role in the mitochondrial pathway of necrosis. The opening of MPTP, caused by intracellular Ca^2+^, inorganic phosphate, alkaline pH, and reactive oxygen species (ROS), leads to mitochondrial membrane permeability, mitochondrial depolarization, loss of proton gradient, and the cessation of ATP production. Therefore, mitochondrial swelling, outer membrane rupture, and simultaneous destruction of the cell membrane and organelles occur; all events that characterize the mitochondria-mediated pathway of necrosis [27].

Necrosis can also be activated through the death receptor pathway. This pathway is induced by the same ligands that activate apoptosis, such as TNF-α, the Fas ligand, and TNF-related apoptosis-inducing ligand. A key role is played by receptor-interacting proteins (RIPs). The binding of TNF-α to TNF receptor 1 (TNFR1) determines the exposure of the death domain of TNFR1 in the cytoplasm. This leads to the recruitment of TNF-receptor-associated death domain (TRADD), which constitutes a scaffold for the assembly of complex I (composed of TNFR1, TRADD, RIP1, TNF-related apoptosis-inducing ligand receptors (TRAF2), and cellular inhibitors of apoptosis 1/2 (cIAP1/2)) on the plasma membrane. NF-κB is activated by this complex and, in turn, activates a cell self-rescue pathway by inhibiting the activation of downstream caspases. Phosphorylation of the necrotic bodies formed by RIP1 and RIP3 activates downstream catabolic enzymes, increases oxidative phosphorylation, and produces ROS, leading to necrosis. Phosphorylated RIP1/RIP3 also activates the mixed-lineage kinase domain-like protein (MLKL) [28,29], which triggers cell and organelle destruction, alters cell permeability, and induces necrosis [30] (Figure 1).

### 2.2. Pro-Inflammatory Apoptosis: Pyroptosis and Its Role in the Pathogenesis of Hepatocarcinoma

Pyroptosis, the caspase-dependent trigger of systemic inflammation via apoptotic cell death, is often classified into classical and nonclassical pathways [31,32]. In both pathways, respectively activated by the cysteine-containing proteases caspase-1 and caspase-4, caspase-5, and caspase-11, the cleavage of gasdermin D (GSDMD) is executed [31,33]. Once the C-terminal domain of GSDMD, which exerts autoinhibitory activity, is detached, the N-terminus leads to the creation of a pore into the plasma membrane with consequent cell swelling and generation of large membrane blebbing, ruptures, and release of cell content culminating in cell death [31,34]. The activation of pyroptosis by the executive-apoptotic protein caspase-3 has also been reported to trigger GSDME cleavage [35,36].

Interestingly, the caspase-3/GSDME signaling pathway is a “switch” that can shift the balance between apoptosis and pyroptosis in cancer. GSDME is cleaved by caspase-3 when it is highly expressed to trigger pyroptosis; otherwise, it triggers apoptosis [35,37].

Like GSDMD, the cleavage of GSDME can degrade the autoinhibitory structure generated by the binding of N-terminal and C-terminal domains, generating a fragment known as the N-terminal domain of GSDME (GSDME-N). GSDME-N penetrates the plasma membrane and guides the conversion of noninflammatory apoptosis into a fast inflammatory pyroptosis [35,36]. Being pivotal substrates for pyroptotic cell death, GSDMD and GSDME are crucial in mounting different types of pyroptotic process that, regardless of the types of pore formation, trigger the release of specific cytokine subsets, including interleukin (IL)-1β and IL-18 [31,38]. IL-18 is a proinflammatory cytokine that mediates the immune response of natural killer (NK) cells and polarized T helper 1 (Th1) cells. Inactive IL-18 precursors are cleaved by caspase-1 for their activation and can stimulate interferon γ and regulate Th1 and Th2 cell-mediated responses [14]. Of note, GSDME-N has been shown to preferentially permeate mitochondria and to subsequently induce ruptures into the plasma membrane [14]. These events trigger pore formation and enable the release of mtDNA as a DAMP-secreted molecule at the extracellular level upon the coordinated pore formation and rupture of mitochondrial and plasma membranes [14]. GSDME-N can also induce fragmentation of the mitochondrial network during pyroptosis and apoptosis [39] (Figure 1).

Although several studies indicate that pyroptosis plays a crucial role in the development of systemic inflammatory syndromes, its contribution has also been recognized in the development and eventual treatment of tumors [39,40,41,42]. Pyroptosis programmed cell death, other than being pivotal in embryonic development, is involved in the onset and progression of several disease conditions, especially cancer. Escaping from cell death is, indeed, a feature of cancer cells [41,42]. Normal cells, when hyperstimulated by inflammatory mediators released by the activation of pyroptosis, may acquire malignant phenotypes [43]. In a recent study, Deng et al. [10] have shown that pyroptotic gene activation can play a significant role in the modulation of tumor immunity and may assist in predicting the prognosis of patients with HCC. Nevertheless, the exact relationship between pyroptosis, HCC development, and patient survival is still unclear.

Even more, the mechanisms through which pyroptosis operates in HCC and in relation with the response to immunotherapy protocols in liver cancer are missing. Alterations in mitochondrial metabolism and GSDME-driven pyroptosis may be relevant molecular signaling routes through which the delivery of mtDNA as DAMP may operate and exacerbate inflammation, which characterizes this type of cancer. The definition of the molecular alterations occurring in HCC patients and their correlation with immunotherapy response may allow for defining the mechanisms of HCC progression, identifying biomarkers of immunotherapy response, and stratifying HCC etiology for targeted therapy.

The analysis of GSDME expression in the Human Protein Atlas (TCGA database) showed a direct association between its expression and the survival of HCC patients. In particular, a high expression of GSDME was associated with significantly decreased survival compared with low/medium expression. Similarly, analyses by the Ualcan platform showed increased expression of GSDME in cancer tissues compared with normal counterparts and a tumor-grade-dependent increase. Finally, the analysis of the inflammatory status and the characterization of circulating mononuclear cells and intestinal permeability in patients with nonalcoholic fatty liver disease (NAFLD)-related cirrhosis with and without HCC revealed significant associations and provided insights on their involvement in hepatocarcinogenesis [44]. A model depicting the most relevant correlations among all measured parameters was built, showing that increased levels of fecal calprotectin characterized patients with HCC, while intestinal permeability was similar to that of patients with cirrhosis but without HCC [44]. Furthermore, an increase in plasma levels of IL-8, IL-13, chemokines (C-C motif), ligands (CCL) 3, CCL4, and CCL5 was observed in patients with NAFLD-related cirrhosis with HCC and was associated with an activated status of circulating monocytes. These findings indicate that, in patients with cirrhosis and NAFLD, systemic inflammation is associated with the process of hepatocarcinogenesis [44].

## 3. Mitochondria-Derived Vesicles in Hepatocarcinoma: Knowing the Unknown

Mitophagy is the process through which damaged mitochondria are recycled, thus representing an essential housekeeping mechanism for preserving mitochondrial quality [45]. However, its assessment in vivo is hampered by technical constraints that also hinder translational applications [45]. The endosomal–lysosomal system is part of the cellular membranous system participating in the endocytic pathway that internalizes, recycles, and modulates various cargo molecules and organelles essential for normal cellular functions. This pathway is a relevant and more accessible component of the mechanisms that maintain cell quality.

EVs have been identified as a highly heterogeneous population of vesicles released into cell culture media and/or biofluids (e.g., plasma, serum, urine, saliva) with wide ranges of size, function, and biogenesis [46]. Subpopulations of EVs with different origins and holding different physical and biochemical features exist [46]. One major classification of EVs is between exosomes and ectosomes [47]. Ectosomes originate from the outward budding of the plasma membrane and have a diameter of 100–500 nm. Ectosomes include exosome-like vesicles, nanoparticles, microparticles, microvesicles, shedding vesicles, and oncosomes [48]. According to the International Society of Extracellular Vesicles (ISEV) guidelines, exosomes refer to EVs of endosomal origin and with a diameter of 50–150 nm [48]. Exosomes are an evolution of intraluminal vesicles (ILVs) generated from an inward budding of the membrane of early endosomes that subsequently originate multivesicular bodies (MVBs) [49,50]. These MVBs/late endosomes usually purse a progressive acidification to finally reach the stage of mature lysosomes. Herein, vesicle cargoes are degraded. As an alternative pathway, MVBs can approach the plasma membrane, fuse, and release their ILVs, which become exosomes, within the extracellular space [5,51]. The thorough characterization of exosomes is not an easy task because the definition of EV biogenesis, according to the ISEV guidelines, could only be ascertained via live imaging assays capturing EV release [52]. This clearly represents a technical constraint for most studies, especially those conducted on humans, and implies that most investigations are conducted on populations of exosomes and are biased by lack of purity of EVs. For this reason, the use of the generic term EVs is preferred when not referring to a pure exosome population, because EVs would also include exosomes, as indicated by the ISEV [48].

In recent years, a particular subset of EVs, called MDVs, has been indicated as an informative readout of mitochondrial function and signaling and a potential surrogate measure of mitochondrial quality [53]. Several studies have reported mitochondrial signatures in EVs in relation to aging and associated conditions (e.g., physical frailty and sarcopenia, neurodegeneration) for which MDVs have been indicated as surrogate circulating markers of cellular quality decline [7,54,55,56,57]. These conditions are all marked by mitochondrial dysfunction and altered mitochondrial quality control (MQC) mechanisms. MDVs are considered, in fact, part of the MQC signaling pathways [58,59] being generated to eject single damaged mitochondrial constituents to circumvent mitophagy and, therefore, proceed with mitochondrial elimination only upon irreversible organelle failure. The formation of MDVs is enacted under physiological conditions and proceeds at basal level, but it increases during pathological stress [60,61]. In particular, the damage of proteins, lipids, and nucleic acids under high ROS generation triggers MDV formation to promote the clearance of oxidized and dysfunctional mitochondrial particles [61,62]. During mild oxidative stress, instead, local activation of phosphatase and tensin homologue (PTEN)-induced putative kinase 1 (PINK1)/parkin occurs. This results from the oxidation of mitochondrial membrane proteins with the consequent budding of oxidized membrane proteins into vesicles [63,64]. When a complete depolarization and organelle dysfunction ensue, the mechanism of MDV biogenesis switches, by means of signals that have not been completely deciphered, towards mitophagy [63].

Under physiological conditions, PINK1 crosses the mitochondrial import channel continuously to enter the mitochondrion. Subsequently, PINK1 is delivered to the cytoplasm, cleaved, and rapidly degraded, while parkin resides in the cytosol in its inactive autoinhibited E3 ubiquitin-ligase [65]. During mild stress conditions, mitochondrial import channels are disrupted and, here, the import process is blocked because of the PINK protein stalling at the level of the import channel or at the outer membrane. Next, ubiquitin and the ubiquitin-like domain of parkin are phosphorylated by PINK1, determining the stabilization of the active parkin protein, and finally assisting MDV generation and release. The biogenesis of MDVs requires Rab9 (a small GTPase regulating vesicle trafficking towards the endo-lysosomal compartments) and vesicular sorting protein sorting nexin 9 (SNX9), although the process regulating their trafficking is unclear [63]. Once generated, MDVs join MVBs to enable their fusion with lysosomes for degradation of oxidized mitochondrial constituents [62]. In this regard, MDVs have been attributed a role as a first line of defense for their ability to expel damaged mitochondrial constituents as a rescue strategy before achieving mitochondrial failure and mitophagy-guided elimination. Via this mechanism, according to cellular demands, the mitochondrial proteome (composed of >1000 proteins) can be preserved as well as functional mitochondrial integration [60,66,67,68,69]. For instance, cancer cells and other types of cells in which mitophagy is deficient use the release of MDVs and the subsequent lysosomal degradation of damaged mitochondrial particles as a compensatory and adaptive mechanism to achieve mitochondrial health [70,71,72]. MDVs are also engaged in mitochondrial turnover via their ability to assist degradation of damaged cargo and enable its replacement with novel and functional proteins and lipids. Importantly, MDVs are also implicated in inter-organellar crosstalk, which is an additional evolutionary conserved trait of mitochondria [73,74].

To this purpose, we have recently shown that synergistic defects of mitochondrial and lysosomal functions, with the consequent inability to degrade MDV cargo and activate mitophagy, determine an increase in MDV secretion as a mechanism of mitochondrial quality check and intercellular communication [75].

Patients with NAFLD show mitochondrial dysfunction and oxidative stress [76]. Several degrees of ultrastructural mitochondrial alterations have been identified in fatty liver disease, including changes in mitochondrial morphology, deficits in electron transport chain (ETC) activity, ATP depletion, increased permeability of the outer and inner mitochondrial membranes, overproduction of ROS and associated increase of oxidative stress, deletions in the mtDNA, and impairment in mitochondrial β-oxidation [77,78]. Recent investigations have indicated that mitochondrial dysfunction and liver senescence are major contributors to the development and progression of NAFLD [79]. Nevertheless, the underlying molecular mechanisms are not fully deciphered. It is known that reduced ETC activity along with higher fatty acid oxidation and ROS levels favors insulin resistance. This, combined with the delivery and accrual of free fatty acid into the liver, predisposes to the pathogenesis of NAFLD [76].

A recent study in humans supported the existence of a link between mitochondrial decline and NAFLD/nonalcoholic steatohepatitis (NASH) development, showing a tight association between NAFLD/NASH, compromised mitochondrial fatty acid oxidation in the liver, and reduced hepatic mitochondrial quality due to impaired organelle turnover [80]. The authors observed a decrease of 40–50% of hepatic mitochondrial complete fatty acid oxidation in patients with NASH compared with controls and normal liver histology [80]. This reduction was paralleled by an increase in hepatic mitochondrial ROS production and reduced mitochondrial biogenesis and mitophagy [80]. The results from this study also indicate that impaired hepatic fatty acid oxidation and reduced mitochondrial turnover are associated with increasing NAFLD severity in obese individuals.

Higher rates of mtDNA mutations, including those affecting genes encoding for ETC complexes, were found in NAFLD patients, with an increase in the mutational rate according to the severity of liver histopathological abnormalities [81]. Mutations in genes encoding ETC subunits were also associated with a reduced gene expression [81]. The activity of the mitochondrial respiratory chain complexes was found to be reduced in liver tissue of animal models and patients with NAFLD [82,83]. In particular, patients with NAFLD showed a reduction of respiratory chain activity of about 37% in complex I, 42% in complex II, 30% in complex III, 38% in complex IV, and 58% in complex V compared with controls without NAFLD [82].

**Figure 2:**
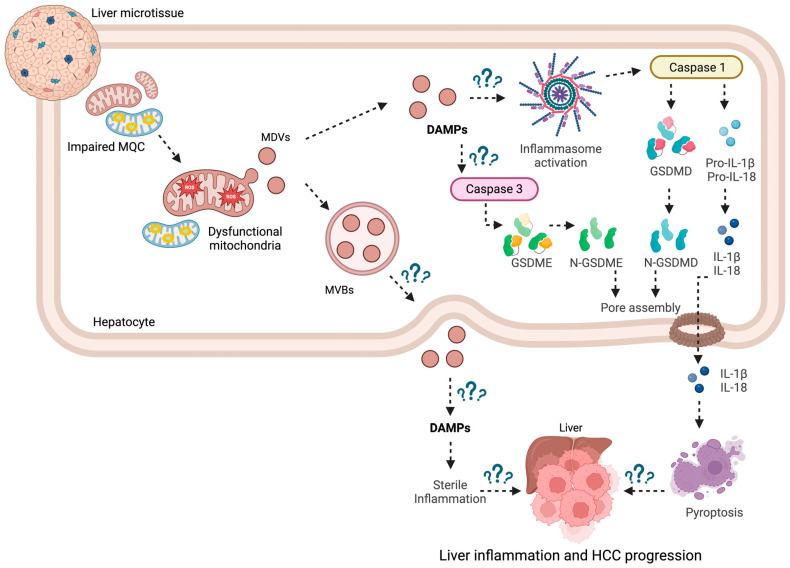
Schematic representation of the molecular routes linking mitochondrial quality control impairment, generation of mitochondria-derived vesicles, and pyroptosis in hepatocarcinoma. Abbreviations: DAMPs, damage-associated molecular patterns; GSDM, gasdermin; HCC, hepatocarcinoma; IL, interleukin; MDVs, mitochondria-derived vesicles; MQC, mitochondrial quality control; MVBs, multivesicular bodies. Created with BioRender.com (accessed on 24 April 2024).

These observations have led to the hypothesis that a similar mechanism could also be in place in liver cancer where cells may use MDV secretion as an MQC mechanism, but also as a pathway to communicate with the tumor microenvironment (Figure 2). However, very few studies have addressed these aspects and most of them focused on EV secretion in HCC as delivery systems for miRNA cargoes, to regulate in recipient cells target genes involved in proliferation, invasion, metastasis, and apoptosis [84]. For instance, the levels of exo-miR-21 were found to be higher in patients with HCC compared with patients with chronic hepatitis B or healthy individuals and had a higher diagnostic sensitivity [85].

Furthermore, it has been shown that loss of exosomal exo-miR-320a from cancer-associated fibroblasts (CAFs) in HCC can induce a downstream extracellular signal-regulated kinases-mediated activation of receptor cells (hepatocytes), leading to lung metastasis [86]. Similarly, the release by CAF of miR-1247-3 encapsulated in exosomes can induce lung metastasis of HCC [87]. Other studies demonstrated that the invasion of hepatoma cells was promoted by macrophages through the secretion of exosomes containing exo-miR-92a-2-5p [88]. Exo-miR-23a/b can be secreted by adipocytes and transported to cancer cells via exosomes, thereby supporting HCC cell growth and migration [89]. Furthermore, exosomes released by cancer cells can contribute to tumor proliferation and dissemination. Some researchers have hypothesized that the acidic tumor microenvironment induces HCC to release exo-miR-21 and exo-miR-10b via exosomes to promote the propagation and metastatization of cancer cells. In light of the multiple roles attributed to miRNAs, these circulating molecules may serve as prognostic molecular markers and therapeutic targets of HCC [90]. Additional research is warranted to establish causality between exosome-delivered miRNAs and HCC progression.

A fairly large amount of research has been devoted to understanding the implication of EVs in HCC progression, immune escape, and tumor invasion [91]. However, the role of MDVs, including MDV biogenesis and cargo shuttling, in the biology of HCC is poorly investigated. The characterization of MDVs in this setting, along with the analysis of pyroptosis, may shed light on the possible link between mitochondrial dysfunction and the caspase-dependent trigger of systemic inflammation via apoptotic cell death and their implication in tumor immunity. This knowledge could unveil relevant details on HCC biology, thereby supporting the identification of new druggable pathways and biomarkers for early diagnosis and tracking of the disease.

## 4. From Bench to Bedside: Clinical Management of Hepatocarcinoma and Immunotherapy

The clinical management of HCC currently relies on therapeutic strategies ranging from liver transplantation to tumor ablation with different methods. The latter include either liver resection and locoregional therapies, such as trans-arterial chemoembolization (TACE) and radioembolization (TARE) [92], or systemic therapies in the setting of advanced and unresectable tumors [92]. Several factors must be considered when choosing the best treatment strategy. Among these are tumor burden, the stage of liver disease, and the degree of portal hypertension [93]. However, the overall patient’s fitness and performance status, the presence of comorbidities, personal preferences, and social support are relevant factors in decision-making [93]. Based on these considerations, the paradigm of HCC management has recently shifted to a multiparametric therapeutic hierarchy [94]. Accordingly, the most suitable choice is established based on potential evidence-based efficacy and feasibility for each patient after a multidisciplinary evaluation, rather than according to a more rigid staging and treatment system based on the algorithm proposed by the Barcelona Clinic Liver Cancer (BCLC) criteria [95]. Another concept closely linked to this new approach is the possibility of a converse strategy, which means that therapies placed downstream in the hierarchy could be used for downstaging the tumor and possibly allow clinicians, in case of a good response, to reconsider treatments that are upstream in the same hierarchy, with better survival benefit.

The diagnosis of HCC is often reached at an advanced stage of the disease, when systemic therapy is the only suitable treatment option. HCC is known to be resistant to traditional cytotoxic chemotherapy, so that other systemic therapies have been developed in recent years. The first ones are tyrosine kinase inhibitors (TKIs), like sorafenib [96] and lenvatinib [97], that have been shown to improve overall survival compared with placebo in first-line treatment, while other TKIs, namely regorafenib [98] and cabozantinib [99], and the monoclonal antibody ramucirumab targeting the vascular endothelial growth factor receptor 2 [100], have been approved in the second-line setting.

**Figure 3:**
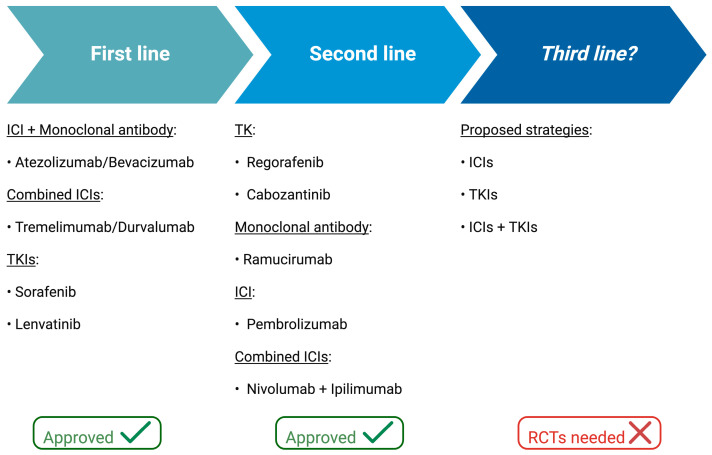
Schematic representation of treatment lines for hepatocarcinoma. Abbreviations: ICIs, immune checkpoint inhibitors; RCTs, randomized clinical trials; TKIs, tyrosine kinase inhibitors. Created with BioRender.com (accessed on 24 April 2024).

Immune checkpoint inhibitors (ICIs) have recently been introduced in the therapeutic scenario of advanced HCC. In particular, the combination of atezolizumab (an anti-programmed death-ligand 1 (PD-L1) agent) and the monoclonal antibody bevacizumab, an anti-vascular endothelial growth factor (VEGF), showed better overall survival (19.2 vs. 13.4 months) and progression-free survival (6.9 vs. 4.3 months) compared with sorafenib in the first-line setting [101,102]. Therefore, as of today, atezolizumab plus bevacizumab has become the first-line treatment of choice. Recently, another association based on two ICIs, the anti-PD-L1 agent durvalumab plus the anti-cytotoxic T-lymphocyte antigen 4 (CTLA-4) agent tremelimumab, has been shown to lead to a better overall survival than sorafenib (hazard ratio 0.78; 95% confidence interval, 0.65–0.92) [103], and has been approved by the FDA in the first-line setting. Additionally, pembrolizumab, an anti-PD1 (programmed death 1) agent, and the association of nivolumab (anti-PD1) plus ipilimumab (anti-CTLA-4) have been approved in the second-line treatment of HCC [104,105] (Figure 3).

Finally, HCC etiology is also a relevant factor to be considered in therapeutic decision-making. Results from the analysis of NASH-affected livers from preclinical models revealed a progressive accrual of inefficient and unconventionally activated CD8 + PD1 + T cells. Furthermore, both preclinical models of NASH-induced HCC and human samples with NAFLD or NASH under immunotherapy treatment targeting PD1 exhibited an expansion of activated CD8 + PD1 + T cells within the tumor that indicated a compromised tumor immune surveillance [106]. The same anti-PD1 treatment in mouse models of CD8 + T cells’ depletion or TNF-guided neutralization induced an increase in the incidence of NASH-related HCC and tumor nodules (number and size). These data suggest that CD8 + T cells support NASH-induced HCC, rather than invigorating or enacting immune surveillance. Indeed, a meta-analysis of three randomized phases III clinical trials revealed that immune therapy did not improve survival in patients with non-viral HCC [106]. However, patients with NASH-induced HCC receiving anti-PD1 or anti-PDL1 had reduced overall survival compared with HCC patients with other etiologies [106]. Therefore, non-viral HCC, and especially NASH-induced HCC, may be less responsive to immunotherapy programs based on anti-PDL1 and anti-VEGF [106] treatments. This may be due a dysregulated NASH-associated T cell activation that may induce tissue damage and lead to altered immune surveillance. These pilot findings indicate that the characterization of the molecular events that follow immunotherapy may help define individual patient responses to this treatment regimen and identify possible adjuvants for ICIs. In this context, it is not negligible to consider that HCC tumorigenesis is associated with persistent inflammation [1].

Inflammatory mediators have also been attributed a role as biomarkers capturing the relationship between tumor microenvironment and an individual’s immune response [107,108,109] and may have a prognostic role in predicting recurrence and mortality after tumor resection. Indices like lymphocyte-to-monocyte ratio, lymphocyte-to-C reactive protein ratio, neutrophil-to-lymphocyte ratio, and platelet-to-lymphocyte ratio have been shown to predict overall and disease-free survival in the setting of TACE or surgical resection [107,110]. However, no consensus has been reached on cutoff values for these scores, which warrants investigation for their implementation in clinical practice. Validation of these biomarkers may assist in patient selection for transplantation and reduce the risk of post-transplant recurrence.

In addition to systemic inflammation, chronic sterile inflammation ensues after the bulk release of molecules, collectively indicated as DAMPs, upon tissue injury [5], of which a thorough characterization is much needed. No major achievements have been reached on the identification of diagnostic and prognostic biomarkers of HCC. The definition of EV biogenesis in HCC may help unequivocally identify EV of hepatic derivation in biological fluids. Moreover, the analysis of their cargoes, in terms of nucleic acids, proteins, and mediators of inflammation, may allow identification of markers of choice for the diagnosis and prognosis of HCC. In this regard, liquid biopsy approaches, including the analysis of EVs, would be of utmost importance owing to their minimal invasiveness and predictive value, which may help monitor HCC progression and facilitate its therapeutic management.

Future clinical research should explore the molecular signatures of HCC linked to different etiologies of liver disease to allow a more personalized treatment approach. This knowledge would eventually allow new drugs with specific targets to be developed and treatment strategies to be devised based on the precision medicine paradigm. Another major goal to be accomplished is the investigation of molecular markers to predict treatment response, especially with regard to immunotherapy, which is currently an unmet need.

## 5. Conclusions

MDVs, a subpopulation of EVs and a potential surrogate marker of mitochondrial quality, are a missing link in HCC biology. An integrated characterization of MDVs and inflammation can help understand the molecular mechanisms regulating HCC onset and progression. EVs have been implicated in HCC growth, metastasis, angiogenesis, and immunosurveillance escape. Pyroptosis, the caspase-dependent trigger of systemic inflammation via apoptotic cell death and a relevant player in tumor immunity, can also be elicited by mitochondrial displaced components. The comprehensive analyses of these molecular pathways may help identify the missing links between HCC development, mitochondrial quality, and inflammation. This knowledge, in turn, will be instrumental in identifying predictive biomarkers and developing new therapeutics.

## Data Availability

Not applicable.
